# Effect of exercise interventions on glycemic control in women with gestational diabetes mellitus: a systematic review and meta-analysis

**DOI:** 10.4069/whn.2025.08.25.1

**Published:** 2025-09-30

**Authors:** Jihoo Her, Myung-Haeng Hur

**Affiliations:** 1 College of Nursing, Eulji University, Uijeongbu, Korea; 2 Department of Nursing, Gimcheon University, Gimcheon, Korea

**Keywords:** Exercise, Gestational diabetes, Glycemic control, Meta-analysis, Systematic review

## Abstract

**Purpose:**

This study aimed to examine the overall impact of randomized controlled trials (RCTs) assessing the effects of exercise interventions—considering program duration, timing, and type—on blood glucose control in women with gestational diabetes mellitus (GDM), thereby improving the reliability of evidence in this field.

**Methods:**

For this systematic review and meta-analysis, we searched major international and domestic databases (PubMed, Embase, Cochrane, CINAHL, RISS, DBpia, NSDL, and KISS) for RCTs published up to December 2024 in English or Korean. Participants were pregnant women diagnosed with GDM. Interventions involved exercise for blood glucose management, while the control group received routine care. The outcome variables were blood glucose levels, including fasting blood sugar (FBS), 2-hour postprandial glucose (PPG2hr), and glycated hemoglobin (HbA1c). Fifteen studies were selected and analyzed using a random-effects model, with mean difference (MD) and 95% confidence interval (CI).

**Results:**

Glycemic parameters in the exercise group improved significantly compared with those in the routine care group: FBS, –0.47 mmol/L (n=963; MD=–0.47; 95% CI, –0.69 to –0.24; *p*<.001), PPG2hr, –0.62 mmol/L (n=944; MD=–0.62; 95% CI, –0.84 to –0.40; *p*<.001), and HbA1c, –0.39% (n=259; MD=–0.39; 95% CI, –0.53 to –0.25; *p*<.001).

**Conclusions:**

Exercise intervention is an effective strategy for regulating blood glucose levels in women with GDM. Moreover, engaging in exercise approximately 15 minutes after meals and scheduling sessions 7 to 10 times per week may be more effective than current recommendations of at least 30 minutes of exercise three times weekly.

## Introduction

Gestational diabetes mellitus (GDM), a common complication during pregnancy, is defined as diabetes first diagnosed between approximately 24 and 28 weeks of gestation and is caused by hormonal and physiological changes during pregnancy [1,2]. The global prevalence of GDM is estimated at about 14% of pregnant women worldwide [3].

Women with GDM face increased risks to both maternal and fetal health, including a higher likelihood of fetal malformations, premature birth, infections, elevated fetal mortality, and a greater probability of cesarean section [4,5]. Even after delivery, women with GDM are at higher risk of developing type 2 diabetes, cardiovascular disease, and hypertension compared with healthy pregnant women [6,7]. Therefore, proper blood glucose management is essential to protect both maternal and fetal health and to minimize complications [8].

For women with GDM, lifestyle interventions such as blood glucose self-monitoring, dietary management, and exercise represent the first-line treatment [8]. These strategies alone successfully manage 70%–85% of cases [9]. The primary goals of exercise in GDM are to maintain normal blood glucose levels and manage weight, thereby reducing complications [2,8]. Some earlier studies suggested an association between exercise and increased risk of preterm labor, attributed to catecholamine release and reduced placental circulation [10,11]. However, other studies have shown that exercise during pregnancy does not raise the risk of preterm labor or cesarean section. On the contrary, exercise can prevent and control obesity, gestational hypertension, premature birth, and GDM itself [12,13].

Except for obstetric problems, current guidelines recommend that women with GDM perform moderate-intensity aerobic exercise five times per week for 30 minutes per session, totaling 150 minutes weekly. Alternatively, simple activities such as walking for 10–15 minutes after meals are advised [8]. Moderate-intensity resistance exercise for 1 hour, 2–3 times weekly, is also recommended [14]. Despite these guidelines, supporting evidence remains limited. Variability in intervention characteristics and inconsistent findings regarding their effects on glycemic control contribute to this gap [15,16], underscoring the need for additional empirical clarity.

Studies have reported inconsistent outcomes of exercise on glycemic control in GDM [17]. For example, moderate-intensity aerobic exercise showed no significant effect [18], while low-intensity activity [19] and high-intensity interval training were effective in women without complications [20]. Resistance exercise demonstrated greater benefits than aerobic exercise in one study [7], but generalizability remains limited.

Most earlier reviews have concentrated on the prevention of GDM in otherwise healthy pregnant women [21,22]. Only a few have evaluated exercise effects in women already diagnosed with GDM, and their findings have been inconsistent. One meta-analysis reported significant benefits in women with GDM or at high risk [15], whereas another found no significant effects of supervised exercise programs lasting less than 4 weeks [16]. These discrepancies may stem from differences in intervention characteristics (e.g., type, duration, and intensity), participant factors (e.g., age, gestational age), and methodological variations such as glucose measurement approaches (plasma vs. whole blood). Thus, it is essential to clarify the effects of exercise interventions on glycemic control in women with GDM by analyzing outcomes according to intervention characteristics within clearly defined inclusion criteria.

To address this, the present study aimed to synthesize the most up-to-date evidence to identify the independent effects of exercise and determine optimal intervention features. Through a systematic literature review and meta-analysis, we aim to provide integrated insights for developing safe and effective exercise programs for women with GDM.

## Methods

### Study design

This review was conducted in accordance with the PRISMA (Preferred Reporting Items for Systematic Reviews and Meta-Analyses) 2020 reporting guidelines [23] and followed the methodological procedures outlined in the Cochrane Handbook for Systematic Reviews of Interventions [24]. The study was registered in the International Prospective Register of Systematic Reviews (CRD42023462263).

### Selection criteria

Studies were included if they met the following criteria: (1) participants: pregnant women diagnosed with GDM during the second or third trimester; (2) intervention: studies implementing exercise interventions for blood glucose control; (3) comparisons: routine care; (4) outcome: blood glucose levels measured before and after exercise interventions; and (5) study design: randomized controlled trial. In cases where the mean gestational age was not specified, studies were still included if it was clearly indicated that participants in the second or third trimester had been recruited. Studies that reported only mean glucose levels or presented results exclusively in figures without numerical data were included in the qualitative synthesis but excluded from the meta-analysis.

The exclusion criteria were as follows: (1) counseling or lifestyle intervention studies without explicit exercise intervention; (2) prevention studies targeting high-risk pregnant women without diagnosed GDM; (3) studies published in languages other than Korean or English; (4) non-randomized or quasi-experimental designs; and (5) studies lacking accessible full texts.

The literature selection process was independently conducted by the authors, and no disagreements occurred.

### Search strategy

Independent literature searches were conducted for studies published up to December 2024 in English or Korean. The databases searched were PubMed, CINAHL, Embase, Cochrane Central Register of Controlled Trials (CENTRAL), National Digital Science Library (NDSL), Korean Studies Information Service System (KISS), DBpia, and Research Information Service System (RISS). To minimize publication bias, grey literature was also searched manually. The search strategy employed MeSH terms and text words, with equivalent text words used across databases. Database-specific subject headings such as EMTREE (EMBASE) and CINAHL Headings were not applied. Boolean operators (“AND,” “OR”) and truncation were used appropriately. Terms for participants included “diabetes,” “gestational[mesh],” “gestational diabetes,” and “GDM.” Terms for interventions included “exercise[mesh],” “physical activity,” “aerobic,” “resistance,” “aqua*,” “swimming,” “running[mesh],” “walking[mesh],” “jogging[mesh],” “yoga[mesh],” and “pilates.” Terms for outcomes included “glycemic control[mesh],” “glucose level,” “blood glucose[mesh],” “blood sugar,” “glycated hemoglobin[mesh],” and “HbA1c.” Keywords related to study design (e.g., “randomized controlled trial”) were intentionally excluded to avoid omitting studies that did not specify design in the title or abstract. Instead, the study design was confirmed during full-text screening.

The researchers independently conducted an initial screening of the selected studies based on titles and abstracts, followed by full-text review. Any disagreements would have been resolved with a third reviewer, though none occurred after discussion. Data coding was also performed independently, and discrepancies were resolved by re-examining the original text.

**Figure 1.**
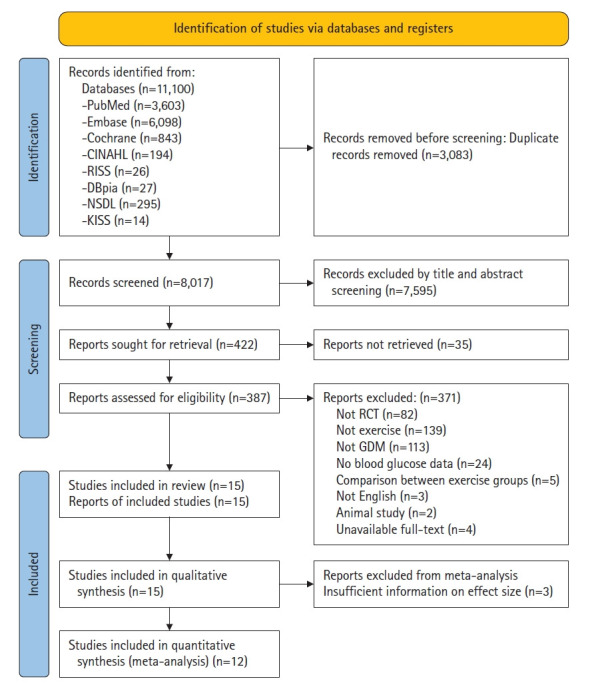
PRISMA 2020 flow chart of study selection process. GDM: gestational diabetes mellitus; RCT: randomized controlled trial.

Using this strategy (Supplementary Material 1), we identified 11,100 studies. After removing 3,083 duplicates and excluding 7,595 irrelevant records, 35 studies without accessible full texts were also excluded. Following full-text review of the remaining 387 studies, 371 did not meet the inclusion criteria. Ultimately, 15 studies [25-39] were included in the qualitative synthesis, of which 12 [25,27-34,36,37,39] were included in the meta-analysis (Figure 1).

### Data extraction

Participant characteristics and intervention details were extracted using Excel (Microsoft Corp., Redmond, WA, USA), including: first author, country, age, pre-pregnancy body mass index, gestational age, sample size, exercise type, main outcome measure, baseline outcome, post-intervention outcome, and author’s conclusion. The primary variables were glycemic outcomes (fasting blood sugar [FBS], 2-hour postprandial glucose [PPG2hr], and glycated hemoglobin [HbA1c]). Effect sizes were calculated using means and standard deviations. Because all outcomes were reported in consistent units, the mean difference (MD) was calculated using the mean values of the intervention and control groups. This review focused exclusively on primary glycemic outcomes; no secondary outcomes were analyzed. Any disagreements during data extraction were resolved through discussion and consensus.

### Methodological quality assessment

Risk of bias in included studies was independently assessed using the Cochrane Risk of Bias 2 (RoB2) tool [40], with discrepancies resolved through discussion. RoB2 evaluates five domains: randomization process, deviations from intended interventions, missing outcome data, outcome measurement, and selection of the reported result. Each domain was assessed using signaling questions, and responses were categorized as “yes,” “probably yes,” “probably no,” “no,” or “no information” [40,41]. Results were visualized using the Robvis tool (https://www.riskofbias.info/welcome/robvis-visualization-tool) and are presented in Supplementary Figure 1.

### Statistical analysis

Analysis, data synthesis, and reporting were conducted according to Cochrane guidelines [24]. Meta-analysis was performed using Review Manager (RevMan, Cochrane, London, UK), ver. 5.4. Regression tests to assess publication bias were conducted in R version 4.3.2 (R Core Team, 2023).

Heterogeneity was evaluated using the Higgins I^2^ statistic. If heterogeneity was indicated (I^2^>50% and *p*>.05), a random-effects model was applied to calculate pooled effect sizes and generate forest plots [42,43]. Publication bias was assessed with funnel plots. Because the funnel plot showed asymmetry, publication bias was considered possible. Therefore, the “meta” and “metafor” packages in R were used [44,45], and Egger’s regression test [46] was conducted to confirm asymmetry.

## Results

For the 12 studies included in the meta-analysis (excluding [26,35,38]), 11 [25,27-32,34,36,37,39] provided sufficient data for effect size analysis of FBS, and 10 [25,27-32,34,36,37] for PPG2hr. Additionally, five studies [30-33,39] enabled effect size analysis of HbA1c. When reviewing intervention characteristics, all studies with available data reported exercise intensity as moderate and frequency as more than three times per week. Accordingly, subgroup analyses were performed for exercise duration, session time, and type.

### Study characteristics

As shown in Table 1, all 15 studies included in the qualitative synthesis were published after 1991, with 10 (66.7%) published from 2010 onward [25-35]. Six studies (40.0%) were conducted in China [26-31]; three (20.0%) in the United States [37-39]; and one each in Brazil [35], Canada [36], Italy [32], Thailand [34], Turkey [25], and Austria [33].

Regarding intervention duration, eight studies (53.3%) reported <8 weeks [27,29,30,33,35,37-39], four (26.7%) reported ≥8 weeks [25,32,34,36], and three (20.0%) provided no or other information [26,31,38]. For exercise type, aerobic activity was reported in seven studies (46.7%) [26,30,32,33,37-39], resistance training in three (20.0%) [27,35,36], and combined exercise in two (13.3%) [25,29], while three (20.0%) provided no or other information [28,31]. For session length, ≥30 minutes was reported in seven studies (46.7%) [26,27,30,33,35,37,38], <30 minutes in four (26.7%) [29,32,34,39], and no information in four (26.7%) [25,28,31,36]. In terms of intensity, moderate-intensity exercise was described in eight studies (53.3%) [26,27,29,30,32,35-37], low intensity in one (6.7%) [38], and six (40.0%) provided no or other information [25,28,31,33,34,39]. Regarding frequency, one study (6.7%) reported<3 sessions per week [34], 12 (80.0%) reported ≥3 sessions weekly [25-27,29,30,32,33,35-39], and two (13.3%) did not specify [28,31].

### Methodological quality of eligible studies

Overall risk of bias was rated low in seven studies (46.7%) [27-30,34-36], with some concerns in six (40.0%) [25,31-33,38,39], and high in two (13.3%) [26,37]. Domains most frequently rated as low risk included deviations from intended interventions and outcome measurement. The domain most often rated high risk was missing outcome data.

### Effectiveness of intervention studies

#### Effect of exercise intervention on fasting blood sugar in gestational diabetes mellitus

**Figure 2.**
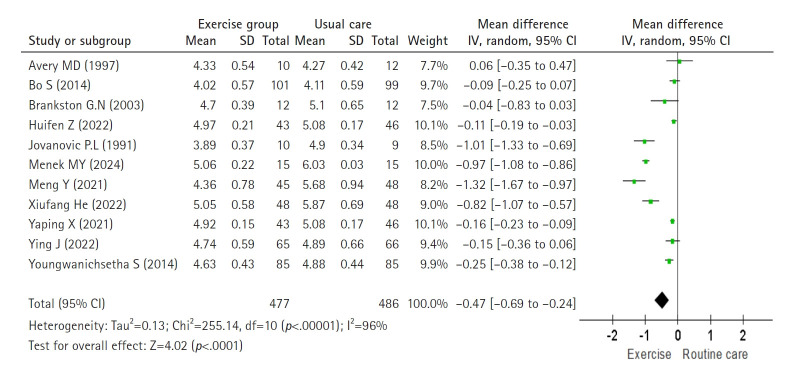
Forest plot of the effect for fasting blood sugar (mmol/L).

The effect of exercise on FBS was analyzed using a random-effects model across 11 studies (Higgins I^2^=96%). Exercise significantly reduced FBS compared with routine care, with a pooled effect size of –0.47 mmol/L (8.47 mg/dL) (n=963; MD=–0.47; 95% CI, –0.69 to –0.24; Z=4.02; *p*<.001) (Figure 2).

##### 1) Effect size of exercise duration on fasting blood sugar

Subgroup analysis by intervention duration showed significant reductions in FBS for both the 1–7 weeks group [27,29,30,37,39] (MD=–0.24 mmol/L [4.32 mg/dL]; 95% CI, –0.42 to –0.07; Z=2.73; *p*=.006) and the not reported group [28,31] (MD=–1.05 mmol/L [18.92 mg/dL]; 95% CI, –1.54 to –0.57; Z=4.23; *p*<.0001). In contrast, the 8–14 weeks group [25,32,34,36] showed a reduction (MD=–0.43 mmol/L [7.75 mg/dL]; 95% CI, –0.91 to 0.05; Z=1.77; *p*=.08), which was not statistically significant. The subgroup difference was significant (*p*=.009) (Supplementary Figure 2).

##### 2) Effect size of exercise time on fasting blood sugar

Subgroup analysis of session time indicated that both the <30 minutes and ≥30 minutes groups significantly reduced FBS compared to routine care. The <30 minutes group [29,32,34,39] showed a MD of –0.34 mmol/L (6.13 mg/dL) (n=520; MD=–0.34; 95% CI, –0.62 to –0.06; Z=2.39; *p*=.02). The ≥30 minutes group [27,30,37] showed a reduction of –0.14 mmol/L (2.52 mg/dL) (n=200; MD=–0.14; 95% CI, –0.19 to –0.09; Z=5.28; *p*<.001). The not reported group [25,28,31,36] also demonstrated significant reductions, with a MD of –0.90 mmol/L (16.22 mg/dL) (n=243; MD=–0.90; 95% CI, –1.16 to –0.64; Z=6.86; *p*<.001). Subgroup differences were statistically significant (*p*<.001) (Supplementary Figure 3).

##### 3) Effect size of exercise type on fasting blood sugar

Subgroup analysis examined the effects of exercise type on FBS. The aerobic exercise group [30,32,37,39] showed a MD of –0.29 mmol/L (5.22 mg/dL) (n=330; MD=–0.29; 95% CI, –0.58 to 0.00; Z=1.94; *p*=.05). The resistance exercise group [27,36] showed a MD of –0.18 mmol/L (3.24 mg/dL) (n=113; MD=–0.18; 95% CI, –0.41 to 0.06; Z=1.45; *p*=.15). The “other exercise” group [25,28,29,31,34] demonstrated an MD of –0.69 mmol/L (12.42 mg/dL) (n=520; 95% CI, –1.10 to –0.28; Z=3.29; *p*=.001). Differences among subgroups were not statistically significant (*p*=.10) (Supplementary Figure 4).

#### Effect of exercise intervention on 2-hour postprandial glucose in gestational diabetes mellitus

**Figure 3.**
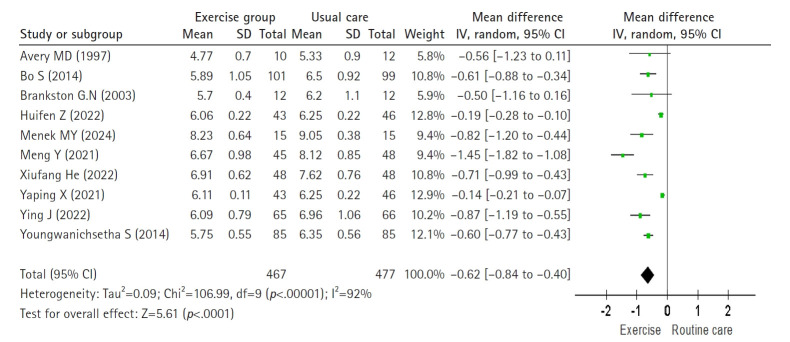
Forest plot of the effect for 2-hour postprandial glucose (mg/dL).

The effect of exercise on PPG2hr was analyzed using a random-effects model including 10 studies (Higgins I^2^=92%). Exercise significantly reduced PPG2hr compared with routine care, with a pooled effect size of –0.62 mmol/L (11.16 mg/dL) (n=944; MD=–0.62; 95% CI, –0.84 to –0.40; Z=5.61; *p*<.001) (Figure 3).

##### 1) Effect size of exercise duration on 2-hour postprandial glucose

The subgroup analysis by intervention duration showed that the 1–7 weeks group [27,29,30,37] demonstrated a MD of –0.33 mmol/L (5.94 mg/dL) (n=331; MD=–0.33; 95% CI, –0.53 to –0.13; Z=3.23; *p*=.001). The 8–14 weeks group [25,32,34,36] showed a larger effect, –0.62 mmol/L (11.16 mg/dL) (n=424; MD=–0.62; 95% CI, –0.76 to –0.49; Z=9.38; *p*<.001). The not reported group [28,31] showed the largest effect size, –1.07 mmol/L (19.26 mg/dL) (n=189; MD=–1.07; 95% CI, –1.79 to –0.34; Z=2.89; *p*=.004). Differences among subgroups were statistically significant (*p*=.02) (Supplementary Figure 5).

##### 2) Effect size of exercise time on 2-hour postprandial glucose

The subgroup analysis by session length showed significant reductions in all groups. The<30 minutes group [29,32,34] showed an MD of –0.65 mmol/L (11.71 mg/dL) (n=501; 95% CI, –0.79 to –0.51; Z=9.00; *p*<.001). The ≥30 minutes group [27,30,37] showed a smaller but significant effect of –0.16 mmol/L (2.88 mg/dL) (n=200; 95% CI, –0.22 to –0.10; Z=5.43; *p*<.001). The not reported group [25,28,31,36] showed an MD of –0.90 mmol/L (16.22 mg/dL) (n=243; 95% CI, –1.29 to –0.51; Z=4.53; *p*<.001). Subgroup differences were statistically significant (*p*<.001) (Supplementary Figure 6).

##### 3) Effect size of exercise type on 2-hour postprandial glucose

Subgroup analysis by exercise type indicated that all categories significantly reduced PPG2hr. The aerobic group [30,32,37] showed a MD of –0.39 mmol/L (7.02 mg/dL) (n=311; MD=–0.39; 95% CI, –0.78 to –0.00; Z=1.98; *p*=.05). The resistance group [27,36] showed –0.20 mmol/L (3.60 mg/dL) (n=113; MD=–0.20; 95% CI, –0.29 to –0.11; Z=4.24; *p*<.001). The “other exercise” group [25,28,29,31,34] demonstrated –0.86 mmol/L (15.48 mg/dL) (n=520; MD=–0.86; 95% CI, –1.13 to –0.60; Z=6.34; *p*<.001). Differences among subgroups were statistically significant (*p*<.001) (Supplementary Figure 7).

#### Effect of exercise intervention on HbA1c in gestational diabetes mellitus

**Figure 4.**
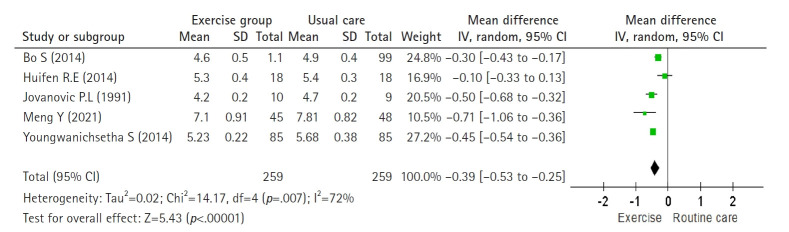
Forest plot of the effect for hemoglobin A1c (%).

Five studies [31-34,39] were analyzed for HbA1c using a random-effects model (Higgins I^2^=72%). Exercise significantly reduced HbA1c compared with routine care, with a pooled effect size of –0.39% (n=518; MD=–0.39; 95% CI, –0.53 to –0.25; Z=5.43; *p*<.001) (Figure 4).

##### 1) Effect size of exercise duration on HbA1c

After excluding one study [16] without information on intervention duration, four studies were analyzed (Higgins I^2^=72%). For interventions<8 weeks [33,39], the effect size showed no statistically significant difference between exercise and routine care: –0.31% (n=55; MD=–0.31; 95% CI, –0.70 to –0.08; Z=1.53; *p*=.120). For interventions ≥8 weeks [32,34], the effect size showed a significant reduction: –0.38% (n=370; MD=–0.38; 95% CI, –0.53 to –0.23; Z=5.10; *p*<.001) (Supplementary Figure 8).

### Publication bias

**Figure 5.**
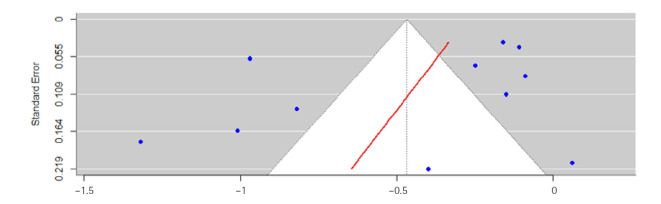
Egger's funnel plot.

As shown in Figure 5, a funnel plot was used to assess potential publication bias, and some asymmetry was observed. Egger’s regression test [46], conducted on 11 of the 12 studies reporting FBS, indicated that the asymmetry was not statistically significant (t=–1.27, *p*=.23).

## Discussion

The results of this meta-analysis of 12 studies demonstrated that FBS, PPG2hr, and HbA1c levels were significantly lower in the exercise intervention group compared with the control group (–0.47 mmol/L, –0.62 mmol/L, and –0.39%, respectively). The glucose control targets for GDM are generally 5.3, 7.8, and 6.7 mmol/L for FBS, PPG1hr, and PPG2hr, respectively [47]. Although evidence on the minimum clinically important difference in glycemic control for women with GDM is still limited [48], previous research has indicated that even a 1 mmol/L change in blood glucose can alter the risk of adverse pregnancy outcomes [49] and is associated with differences in the incidence of cardiovascular disease [50]. Thus, the observed significant reductions in both FBS and PPG2hr in this study can be considered clinically meaningful.

In this meta-analysis, HbA1c was also significantly lower in the exercise intervention group compared with the control group (–0.39%), suggesting that exercise contributes to reducing the risk of GDM-related complications. However, in most included studies, HbA1c levels in both groups were already within the recommended GDM target range of 5.5%–6.5% [51]. This likely reflects the effects of routine care—such as dietary counseling and glucose monitoring—commonly provided to women with GDM. Under these circumstances, while the additional effect of exercise is statistically significant, the clinical interpretation of HbA1c reduction alone requires caution. HbA1c may have limited sensitivity as an outcome measure for exercise interventions in GDM [3]. Since HbA1c reflects average blood glucose over the prior 1–2 months, the duration of exercise, timing of measurement, and attainment of individualized glucose goals should all be considered when evaluating intervention effects.

Nevertheless, prior studies indicate that even small increases in HbA1c (5.1%–5.4% or ≥5.5% vs. ≤5.0%) are significantly associated with increased risks of cesarean delivery, gestational hypertension, and macrosomia [52]. This suggests that modest reductions in HbA1c achieved through exercise may hold clinical significance in GDM management. Therefore, although the absolute reductions observed may appear small, exercise-based glycemic control can help prevent adverse maternal and neonatal outcomes such as macrosomia, as well as long-term metabolic complications.

In the subgroup analyses, the effects of exercise duration showed some variability. For FBS, modest but significant reductions were observed with 1–7 weeks of exercise, while the 8–14 weeks group showed larger but statistically nonsignificant reductions. In contrast, PPG2hr declined more consistently with longer exercise durations, and HbA1c showed significant improvement with 8–14 weeks of intervention. Considering that women with GDM are generally advised to exercise after meals [53], it is reasonable that reductions in PPG2hr were greater than in FBS. Despite the limitations of HbA1c as an outcome measure, the observed decreases suggest that exercise lasting more than 8 weeks may yield greater benefits for glycemic control [25]. Still, due to limited data, further rigorously designed trials are needed to substantiate these findings.

Analysis by session length revealed that both FBS and PPG2hr levels were lower when exercise was performed for 15–29 minutes compared with 30–60 minutes. Women in the shorter-duration group generally exercised more frequently, suggesting that higher frequency may compensate for reduced duration, resulting in superior glycemic outcomes. This aligns with evidence from type 2 diabetes research [53], which found that shorter, more frequent exercise sessions were more effective for glucose regulation. A prior review also noted that even low-intensity exercise, if maintained consistently, can improve insulin resistance [54]. These findings provide scientific support for current recommendations [14] encouraging women with GDM to perform short, frequent bouts of postprandial exercise—such as 10–15 minutes after meals—rather than focusing solely on accumulating 150 minutes per week in sessions lasting ≥30 minutes. The present findings therefore support the recommendation that women with GDM engage in approximately 15 minutes of exercise after meals, 7–10 times per week, rather than limiting activity to three or more longer sessions (30 minutes or more) weekly.

Analysis of the exercise intervention data revealed that FBS improved significantly only with aerobic exercise, while PPG2hr improved significantly regardless of exercise type (aerobic or resistance). Aerobic exercise enhances insulin sensitivity to lower blood glucose levels, whereas resistance exercise increases muscle mass, thereby improving impaired glucose tolerance and glycogen storage capacity [55,56]. Engaging both metabolic pathways simultaneously may be physiologically more beneficial than relying on a single type of exercise [14].

Interestingly, the subgroup categorized as “not reported” consistently showed greater reductions in FBS, PPG2hr, and HbA1c across intervention duration, timing, and type. Although the intervention details in these studies were not explicitly reported, most described individualized exercise programs maintained from enrollment until delivery. The extended duration and tailored nature of these interventions may have contributed to the observed effects. However, because specific program details were unclear and potential confounding factors cannot be ruled out, these findings must be interpreted cautiously in clinical settings. Further rigorous randomized controlled trials (RCTs) are needed to determine whether the observed benefits in this subgroup were attributable to exercise duration, intensity, or individualization.

A key strength of this study is the clearly defined population, consisting solely of women diagnosed with GDM, distinct from those merely at high risk. Compared with prior systematic reviews, this study offers more precise insights by conducting subgroup analyses of detailed exercise parameters—such as timing, duration, and type. These analyses refine individualized recommendations for exercise-based glycemic control in GDM, a topic not fully addressed in earlier reviews. This clarity allowed a more precise identification of the magnitude of glycemic control effects. In addition, a sub-analysis was conducted according to the content of the exercise program and its effects, which enabled the proposal of targeted exercise strategies for GDM management. Moreover, analyzing all three blood glucose indicators (FBS, PPG2hr, and HbA1c) in relation to exercise content may help establish more specific goals for future exercise programs.

Although subgroup analyses were conducted by exercise type, duration, and timing, variables such as exercise intensity, frequency, and measurement methods could not be analyzed because of incomplete and inconsistent reporting across studies. Another limitation was that exercise intensity and frequency were reported in only a few studies, and the reporting formats varied considerably, making direct comparisons difficult. With respect to heterogeneity, overall I^2^ was high for FBS (96%) and PPG2hr (92%) and moderate for HbA1c (72%). By duration (<8 weeks, ≥8 weeks, not reported), I^2^ values were 87%, 97%, and 80% for FBS, and 85%, 0%, and 90% for PPG2hr. By exercise time (15–29 minutes, 30–60 minutes, not reported), I^2^ values were 89%, 0%, and 74% for FBS, and 11%, 4%, and 74% for PPG2hr. By exercise type (aerobic, resistance, not reported), I^2^ values were 90%, 41%, and 96% for FBS, and 83%, 0%, and 77% for PPG2hr. Despite subgrouping by these intervention characteristics, substantial heterogeneity persisted in several subgroups. In particular, the aerobic subgroup showed high heterogeneity, likely reflecting the inclusion of varied exercise modes and intensities under the single category of “aerobic,” thereby reducing homogeneity. Likewise, the “not reported” or “other” subgroups could not be clearly classified, limiting analytical precision. These factors may explain the persistence of heterogeneity. Although some reduction in heterogeneity was observed after subgrouping by exercise timing, considerable variability remained overall. This suggests that unmeasured or unreported factors—such as measurement methods, cultural context, or baseline participant characteristics—may have contributed to heterogeneity. Consequently, these findings should be interpreted with caution. While the funnel plot suggested possible heterogeneity, the Egger test indicated that publication bias was not statistically significant (Figure 5). To address these issues, further RCTs with clear reporting of exercise intensity, frequency, and measurement methods are needed to clarify sources of heterogeneity and more accurately evaluate the effects of exercise interventions. In addition, studies conducted across diverse cultural contexts are warranted.

Accordingly, more rigorously designed experimental studies, together with updated systematic reviews and meta-analyses, are required. The present study supports exercise intervention as an effective strategy for glycemic control in women with GDM. In particular, exercising for approximately 15 minutes after meals, 7–10 times per week, may be more beneficial than the general recommendation of at least 30 minutes of exercise three times per week. These results highlight the potential value of individualized, frequent, and low-burden exercise strategies tailored specifically for women with GDM.

In conclusion, this study found exercise intervention to be more effective when the session length was ≤30 minutes and the intervention period exceeded 8 weeks. Moreover, personalized exercise programs tailored to individual needs may yield additional benefits. Overall, the findings strongly suggest that exercise interventions are effective for improving blood glucose control in GDM, particularly when implemented as short, frequent sessions scheduled after meals.

## Figures and Tables

**Table 1.** Summary of exercise programs in the selected studies (N=15)

First author (year)	Country	Sample characteristics	Intervention (enrolled)	Exercise characteristics	Control group (number)	Measurement methods	Main outcome measures	Baseline	Post-test	Author’s conclusion
Menek (2024) [25]	Turkey	Age (years)	Supervised home exercise (n=15)	D: 8 wk	Routine care (n=15)	Venous	FBS (mg/dL)	I: 106.1±5.6	I: 91.2±4.0	Home exercise with physiotherapy improved glucose and quality of life in GDM.
		- I: 35.1±4.3		TTFI: NR,		Clinician-measured		C:112.9± 3.8	C:108.6±0.6	
		- C: 36.9±2.8		combined (aerobic+ resistance),						
		Pre-intervention BMI: NR		3 times/wk,						
		GA (weeks)		low to moderate						
		-24–28 week								
		(2–3rd trimester)					PPG2hr (mg/dL)	I: 169.7±16.7	I:148.3±11.5	
								C: 169.5± 7.3	C: 163.0±6.8	
He (2024) [26]	China	Age (years)	Aerobic exercise (n=75)	D: NR	Routine care (n=75)	Venous	FBS	NR	NR (only visually confirmed)	Multidimensional exercise improved glucose and delivery outcomes in GDM.
		- I: 29±1.9		TTFI: 30 min,		Clinician-measured				
		- C: 28±1.4		aerobic,						
		Pre-intervention BMI: NR		7 times/wk,						
		GA (weeks)		moderate						
		-24–28					PPG2hr	NR		
		(2nd–3rd trimester)					HbA1c	NR		
Huifen (2022) [27]	China	Age (years)	Resistance exercise (n=43)	D: 6 wk	Routine care (n=46)	Capillary	FBS (mmol/L)	I: 5.3±0.7	I: 5.0±0.2	Moderate resistance exercise improved glucose and insulin use in GDM.
		- I: 31.8±5.2		TTFI: 50-60 min,		Self-monitoring		C: 5.3±0.5	C: 5.1±0.2	
		- C: 31.4± 4.7		resistance,						
		Pre-intervention BMI		3 times/wk,						
		- I: 23.0±5.2		moderate						
		- C: 22.0± 3.0								
		GA (weeks)								
		- I: 28.0±2.0								
		- C: 28.0±2.3					PPG2hr (mmol/L)	I: 6.5±0.5	I: 6.1±0.2	
								C: 6.6± 0.7	C: 6.3±0.2	
Xiufang (2022) [28]	China	Age (years)	Targeted care, exercise intervention	D: NR	Routine care (n=48)	Venous	FBS (mmol/L)	I: 7.0±1.5	I: 5.1± 0.6	Targeted exercise improves glucose and outcomes in GDM.
		- I: 29.0±3.1		TTFI: NR		Clinician-measured		C: 7.0± 1.7	C. 5.9±0.7	
		- C: 28.9±3.7								
		Pre-intervention BMI: NR								
		GA (weeks)								
		- I: 36.2±2:2								
		- C: 36.4±2.4					PPG2hr (mmol/L)	I: 10.1±2.1	I: 6.9±0:6	
								C: 10.1± 2.1	C: 7.6±0.8	
Ying (2022) [29]	China	Age (years)	Gymnastics for pregnant women program	D: 2 wk	Routine care (n=66)	Capillary	FBS (mmol/L)	I: 4.5±0.4	I: 4.9±0.7	Prenatal gymnastics improved glucose control pre- and post-partum.
		- I: 33.5±4.3		TTFI: 15 min,		Self-monitoring		C: 4.5± 0.4	C: 4.7±0.6	
		- C: 32.1±4.4		combined (aerobic+ resistance),						
		Pre-intervention BMI		10 times/wk,						
		- I: 21.3±3.2		moderate						
		- C: 22.2±3.9								
		GA (weeks)								
		- I: 25.7±1.6								
		- C: 25.9±1.4					PPG2hr (mmol/L)	I: 9.1±1.2	I: 6.1± 0.8	
								C: 9.1±1.3	C: 7.0±1.1	
										
Yaping (2021) [30]	China	Age (years)	Aerobic exercise	D: 6 wk	Routine care (n=43)	Capillary	FBS (mmol/L)	I: 5.3±0.6	I: 4.9± 0.2	Moderate aerobic exercise improves glucose control in GDM.
		- I: 31.4± 4.1		TTFI: 50-60 min,		Self-monitoring		C: 5.3±0.5	C: 5.1±0.2	
		- C: 31.4±4.7		aerobic,						
		Pre-intervention BMI		3 times/wk,						
		- I: 23.1±3.7		moderate						
		- C: 22.0±3.0								
		GA (weeks)								
		- I: 28.1±2.0								
		- C: 28.0±2.3					PPG2hr (mmol/L)	I: 6.5±0.4	I: 6.1±0.1	
								C: 6.6±0.7	C: 6.3±0.2	
Meng (2021) [31]	China	Age (years)	Different exercises at different stages (n=45)	D: NR	Routine care (n=48)	NR (FBS, PPG2hr)	FBS (mmol/L)	NR	I: 4.4±0.8	Comprehensive nursing improved maternal and infant outcomes in GDM.
		- I: 26.3±2.0		TTFI: NR		Venous			C: 5.7±0.9	
		- C: 27.0±2.4				Clinician-measured (HbA1c)				
		Pre-intervention BMI: NR								
		GA (weeks)								
		- I: 25.2±4.1					PPG2hr (mmol/L)	NR	I: 6.7±1.0	
		- C: 23.8±3.3							C: 8.1±0.9	
							HbA1c (%)	NR	I: 7.1±0.9	
									C: 7.8± 0.8	
Bo (2014) [32]	Italy	Age (years)	Brisk walking (n=101)	D: 12–14 wk	Routine care (n=99)	Capillary	FBS (mg/dL)	NR	I: 72.4±10.3	In GDM, exercise reduced postprandial glucose but not fasting glucose.
		18–50		TTFI: 20 min,		Self-monitoring			C: 74.1±10.7	
		Pre-intervention BMI		aerobic,						
		- I: 27.6±4.1		7 times/wk,						
		- C: 27.5±4.4		moderate			PPG2hr (mg/dL)	NR	I: 106.1±19.0	
		GA (weeks): 24–26							C: 117.2±16.5	
							HbA1c (%)	NR	I: 4.6±0.5	
									C: 4.9±0.4	
Halse (2014) [33]	Australia	Age (years)	Home-based exercise, cycling (n=20)	D: 6–7 wk	Routine care (n=20)	Capillary	FBS	NR	NR (FBS, PPG2hr only visually confirmed)	Cycling at home may support glucose control in GDM.
		- I: 34±5		TTFI: 45 min,		Self-monitoring				
		- C: 32±3		aerobic,		(FBS, PPG2hr)				
		Pre-intervention BMI		5 times/wk,		Venous, Clinician-measured (HbA1c)				
		- I: 25.2±6.7		low to high						
		- C: 26.4±7.1								
		GA (weeks)								
		- I: 28.8±0.8					PPG2hr	NR		
		- C: 28.8±1.0					HbA1c (%)	NR	I: 5.3±0.4	
									C: 5.4±0.3	
Youngw­anichsetha (2014) [34]	Thailand	Age (years)	Yoga	D: 8 wk	Routine care (n=85)	Capillary	FBS (mg/dL)	I: 88.8±14.5	I: 83.4 ±7.7	Yoga and mindful eating improved glycemic control in GDM.
		- I: 32.6±5.0		TTFI: 15–20 min,		Self-monitoring		C 89.4±14.5	C: 87.9 ±7.9	
		- C: 31.2±4.5		flexibility,		(FBS, PPG2hr)				
		Pre-intervention BMI		1 time/wk,		Venous, Clinician-measured (HbA1c)				
		- I: 27.1±3.6		NR						
		- C: 27.1±4.6								
		GA (weeks)								
		-24–30					PPG2hr (mg/dL)	I: 115.5±7.6	I: 103.7±9.9	
		(2nd–3rd trimester)						C:117.2±12.1	C:114.4 ±10.2	
							HbA1c (%)	NR	I: 5.2 ±0.2	
									C: 5.7 ±0.4	
de Barros (2010) [35]	Brazil	Age (years)	Circuit type resistance (n=32)	D: 6 wk	Routine care (n=32)	Capillary	Mean glucose level (mg/dL)	I: 94.5±23.4	I: 100.3±9.4	Resistance exercise reduced insulin need and improved glycemic control in GDM.
		- I: 31.9±4.9		TTFI: 30–40 min,		Self-monitoring		C: 95.9±14.8	C: 102.9±7.9	
		- C: 32.4±5.4		resistance,						
		Pre-intervention BMI		3 times/wk,						
		- I: 25.3±4.2		moderate						
		- C: 25.4±3.8								
		GA (weeks)								
		- I: 31.6 ±2.3								
		- C: 31.1±2.3								
Brankston (2004) [36]	Canada	Age (years)	Circuit type resistance (n=12)	D: 8 wk	Routine care (n=12)	Capillary	FBS (mmol/L)	I: 4.8±0.6	I: 4.7±0.4	Resistance exercise may help overweight GDM patients avoid insulin.
		- I: 30.5±4.4		TTFI: NR,		Self-monitoring		C: 5.5±0.6	C: 5.1±0.7	
		- C: 31.3± 5.0		resistance,						
		Pre-intervention BMI		3 times/wk,						
		- I: 25.9±3.4		moderate						
		- C: 28.0±5.7								
		GA (weeks)								
		- I: 29.0±2.0								
		- C: 29.6±2.1					PPG2hr (mmol/L)	I: 9.9±1.0	I: 5.7±0.4	
								C: 9.6±1.4	C: 6.2±1.1	
Avery (1997) [37]	United	Age (years)	Cycle ergometer or walking (n=10)	D: 6 wk	Routine care (n=12)	Capillary	FBS (mg/dL)	I: 85±6.8	I: 78±9.8	Partially home-based exercise did not lower blood glucose.
	States	- I: 32.2±4.9		TTFI: 30 min,		Self-monitoring		C: 84±11.7	C: 77±7.6	
		- C: 30.4±5.1		aerobic,						
		Pre-intervention BMI		3 times/wk,						
		- I: 28.4±7.6		moderate						
		- C: 25.5±5.5								
		GA (weeks)								
		- I: 28.7±3.0								
		- C: 26.3±8.1					PPG2hr (mg/dL)	I: 185±18.8	I: 86±12.7	
								C: 187±26.8	C: 96±13.0	
Bung (1991) [38]	United	Age (years)	Bicycle (n=17)	D: 4 wk	Routine care (n=17)	Capillary	Mean glucose level (mmol/L)	NR	I: 5.2±0.3	Moderate exercise safely maintained glucose control in GDM.
	States	- I: 31.0±4.5		TTFI: 45 min,		Self-monitoring			C: 4.92±0.4	
		-C: 32.3±5.7		aerobic,						
		Pre-intervention BMI: NR		3 times/wk,						
		GA (weeks)		mild						
		- I: 30.3±1.9								
		- C: 30.3±2.0								
Jovanovic-Peterson (1991) [39]	United	Age (years)	Arm-ergometer training	D: 6 wk	Routine care (n=9)	Venous	FBS (mmol/L)	I: 5.6±0.5	I: 3.9±0.4	Upper-arm exercise may help manage GDM and reduce insulin need.
	States	- NR	(n=10)	TTFI: 20 min,		Clinician-measured		C: 5.4±0.7	C: 4.9±0.3	
		Pre-intervention BMI: NR		aerobic,						
		GA(weeks) ≥2		3 times/wk,			Mean glucose level (mmol/L)	I: 13.3±1.6	I: 5.9±1.1	
		(3rd trimester)		NR				C: 12.5±1.8	C: 10.4±0.7	
							HbA1c (%)	NR	I: 4.2±0.2	
									C: 4.7±0.2	

BMI, Body mass index (kg/m^2^); C, control group; D, duration; FBS, fasting blood sugar; GA, gestational age; GDM, gestational diabetes mellitus; I, intervention group; NR, not recorded; PPG2hr, 2-hour postprandial glucose; TTFI, time, type, frequency, intensity.

## Supplementary materials

Further details on supplementary materials are presented online (available at https://doi.org/10.4069/whn.2025.08.25.1).

Supplementary Material 1. Search strategy for nine databases

Supplementary Figure 1. Forest plot of the effect of duration for fasting blood sugar.

Supplementary Figure 2. Forest plot of the effect of duration for fasting blood sugar.

Supplementary Figure 3. Forest plot of the effect of time for fasting blood sugar.

Supplementary Figure 4. Forest plot of the effect of exercise type for fasting blood sugar.

Supplementary Figure 5. Forest plot of the effect of duration for 2-hour postprandial glucose.

Supplementary Figure 6. Forest plot of the effect of time for 2-hour postprandial glucose.

Supplementary Figure 7. Forest plot of the effect of exercise type for 2-hour postprandial glucose.

Supplementary Figure 8. Forest plot of the effect of duration for hemoglobin A1c.
