# Supplementary material for: Effect of exercise interventions on glycemic control in women with gestational diabetes mellitus: a systematic review and meta-analysis
Source: Womens Health Nurs. 2025 Sep 30;31(3):176–91. doi: 10.4069/whn.2025.08.25.1 (PMC12571028; doi:10.4069/whn.2025.08.25.1)
Supplement: Supplementary Material 1. — Search strategy for nine databases [file whn-2025-08-25-1-Supplementary-Material-1.pdf]

Supplementary Material 1. Search strategy for nine databases

1.PubMed: 3603 Results
#1(“GDM”[All Fields] OR (“diabetes, gestational”[MeSH Terms] OR (“diabetes”[All Fields] AND “gestational”[All Fields]) OR “gestational diabetes”[All Fields] OR “diabetes gestational”[All Fields]) OR ((“gestate”[All Fields] OR “gestated”[All Fields] OR “gestates”[All Fields] OR “gestating”[All Fields] OR “gestational”[All Fields] OR “gestations”[All Fields] OR “pregnancy”[MeSH Terms] OR “pregnancy”[All Fields] OR “gestation”[All Fields]) AND “diabet*”[All Fields]))
#2 (“exercise”[MeSH Terms] OR “exercise”[All Fields] OR “exercises”[All Fields] OR “exercise therapy”[MeSH Terms] OR (“exercise”[All Fields] AND “therapy”[All Fields]) OR “exercise therapy”[All Fields] OR “exercising”[All Fields] OR “exercise s”[All Fields] OR “exercised”[All Fields] OR “exerciser”[All Fields] OR “exercisers”[All Fields] OR (“exercise”[MeSH Terms] OR “exercise”[All Fields] OR (“physical”[All Fields] AND “activity”[All Fields]) OR “physical activity”[All Fields]) OR (“aerobic”[All Fields] OR “aerobically”[All Fields] OR “bacteria, aerobic”[MeSH Terms] OR (“bacteria”[All Fields] AND “aerobic”[All Fields]) OR “aerobic bacteria”[All Fields] OR “aerobe”[All Fields] OR “aerobes”[All Fields] OR “exercise”[MeSH Terms] OR “exercise”[All Fields] OR “aerobics”[All Fields]) OR (“resist”[All Fields] OR “resistance”[All Fields] OR “resistances”[All Fields] OR “resistant”[All Fields] OR “resistants”[All Fields] OR “resisted”[All Fields] OR “resistence”[All Fields] OR “resistences”[All Fields] OR “resistent”[All Fields] OR “resistibility”[All Fields] OR “resisting”[All Fields] OR “resistive”[All Fields] OR “resistively”[All Fields] OR “resistivities”[All Fields] OR “resistivity”[All Fields] OR “resists”[All Fields]) OR “aqua*”[All Fields] OR (“swimming”[MeSH Terms] OR “swimming”[All Fields] OR “swims”[All Fields]) OR (“running”[MeSH Terms] OR “running”[All Fields] OR “runnings”[All Fields]) OR (“walked”[All Fields] OR “walking”[MeSH Terms] OR “walking”[All Fields] OR “walks”[All Fields]) OR (“jogged”[All Fields] OR “jogging”[MeSH Terms] OR “jogging”[All Fields]) OR (“yoga”[MeSH Terms] OR “yoga”[All Fields]) OR (“pilate”[All Fields] OR “pilates”[All Fields]))
#3 (“glycemic control”[MeSH Terms] OR (“glycemic”[All Fields] AND “control”[All Fields]) OR “glycemic control”[All Fields] OR ((“glucose”[MeSH Terms] OR “glucose”[All Fields] OR “glucoses”[All Fields] OR “glucose s”[All Fields]) AND (“level”[All Fields] OR “levels”[All Fields])) OR (“blood glucose”[MeSH Terms] OR (“blood”[All Fields] AND “glucose”[All Fields]) OR “blood glucose”[All Fields]) OR (“blood glucose”[MeSH Terms] OR (“blood”[All Fields] AND “glucose”[All Fields]) OR “blood glucose”[All Fields] OR (“blood”[All Fields] AND “sugar”[All Fields]) OR “blood sugar”[All Fields]) OR (“glycated hemoglobin”[MeSH Terms] OR (“glycated”[All Fields] AND “hemoglobin”[All Fields]) OR “glycated hemoglobin”[All Fields]) OR (“glycated hemoglobin”[MeSH Terms] OR (“glycated”[All Fields] AND “hemoglobin”[All Fields]) OR “glycated hemoglobin”[All Fields] OR “hba1c”[All Fields] OR “hba1cs”[All Fields]))
#4 #1 AND #2 AND #3
2. Embase: 6098 Results
#1 ‘gdm’ OR ‘diabetes, gestational’/exp OR ‘diabetes, gestational’ OR ((‘diabetes,’/exp OR diabetes,) AND gestational) OR (gestational AND diabet*)
#2 ‘exercise’/exp OR exercise OR ‘physical activity’/exp OR ‘physical activity’ OR (physical AND (‘activity’/exp OR activity)) OR aerobic OR ‘resistance’/exp OR resistance OR aqua* OR ‘swimming’/exp OR swimming OR ‘running’/exp OR running OR ‘walking’/exp OR walking OR ‘jogging’/exp OR jogging OR ‘yoga’/exp OR yoga OR ‘pilates’/exp OR pilates
#3 ‘glycemic control’/exp OR ‘glycemic control’ OR (glycemic AND (‘control’/exp OR control)) OR ‘glucose level’/exp OR ‘glucose level’ OR ((‘glucose’/exp OR glucose) AND level) OR ‘blood glucose’/exp OR ‘blood glucose’ OR ((‘blood’/exp OR blood) AND (‘glucose’/exp OR glucose)) OR ‘blood sugar’/exp OR ‘blood sugar’ OR ((‘blood’/exp OR blood) AND (‘sugar’/exp OR sugar)) OR ‘glycated hemoglobin’/exp OR ‘glycated hemoglobin’ OR (glycated AND (‘hemoglobin’/exp OR hemoglobin)) OR ‘hba1c’/exp OR hba1c
#4 #1 AND #2 AND #3
3. Cochrane: 843 Results
((GDM) OR (diabetes, gestational) OR (gestational diabet*)) AND ((exercise) OR (physical activity) OR (Aerobic) OR (Resistance) OR (aqua*) OR (swimming) OR (running) OR (walking) OR (jogging) OR (yoga) OR (pilates)) AND ((glycemic control) OR (glucose level) OR (Blood glucose) OR (Blood sugar) OR (Glycated hemoglobin) OR (HbA1C)) in Title Abstract Keyword
4. CINAHL: 194 Results
(((GDM) OR (diabetes, gestational) OR (gestational diabet*))) AND (((exercise) OR (physical activity) OR (Aerobic) OR (Resistance) OR (aqua*) OR (swimming) OR (running) OR (walking) OR (jogging) OR (yoga) OR (pilates))) AND (((glycemic control) OR (glucose level) OR (Blood glucose) OR (Blood sugar) OR (Glycated hemoglobin) OR (HbA1C)))
