# Supplementary figures and images for: Effect of exercise interventions on glycemic control in women with gestational diabetes mellitus: a systematic review and meta-analysis
Source: Womens Health Nurs. 2025 Sep 30;31(3):176–91. doi: 10.4069/whn.2025.08.25.1 (PMC12571028; doi:10.4069/whn.2025.08.25.1)

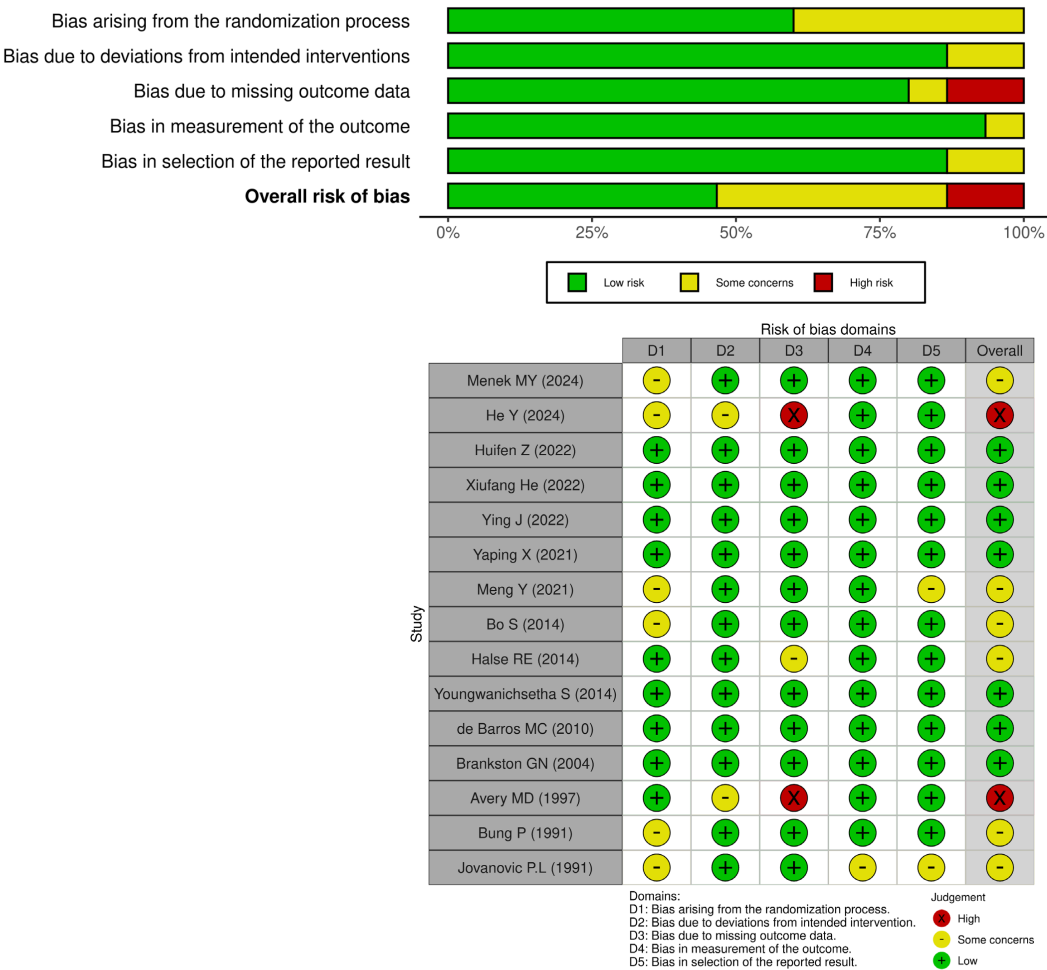

Supplementary Figure 1. Risk of bias in included studies.

Supplement: Supplementary Figure 1. — Forest plot of the effect of duration for fasting blood sugar. [file whn-2025-08-25-1-Supplementary-Figure-1.pdf]

## 1.3 FBS(time)

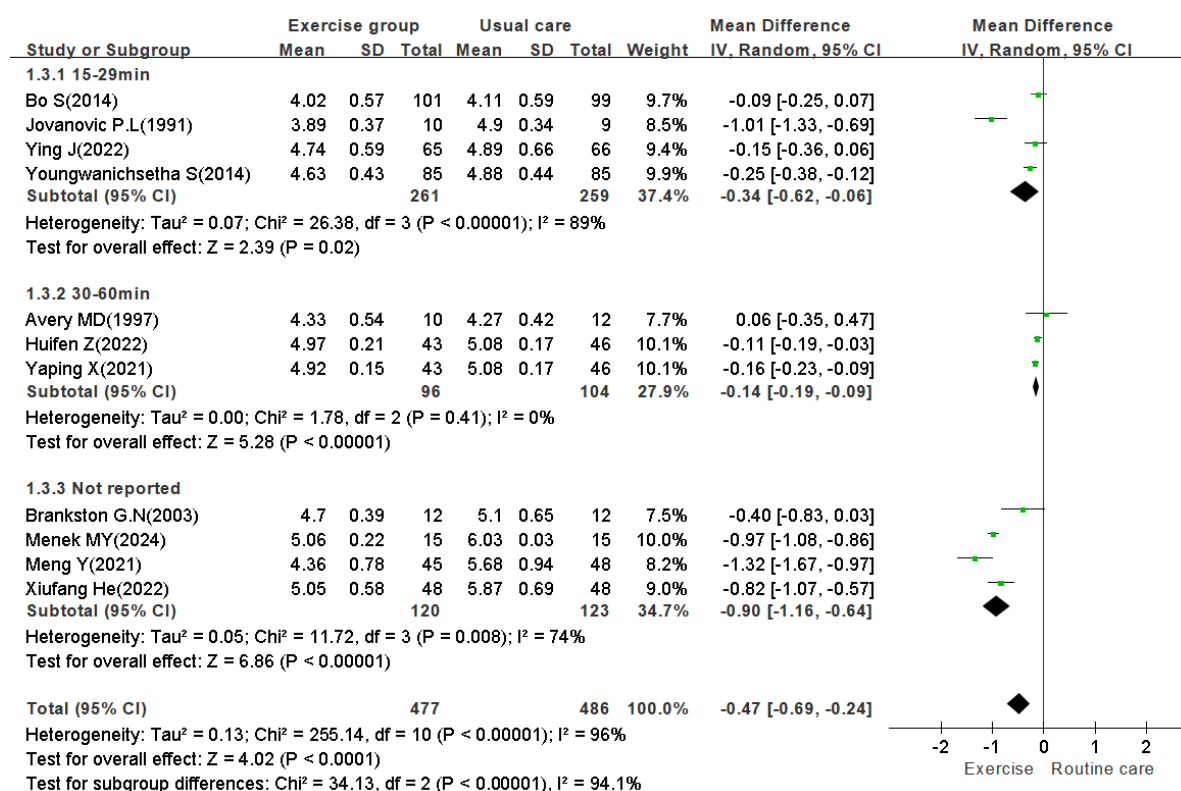

Supplementary Figure 3. Forest plot of the effect of time for fasting blood sugar.

Supplement: Supplementary Figure 3. — Forest plot of the effect of time for fasting blood sugar. [file whn-2025-08-25-1-Supplementary-Figure-3.pdf]

## 1.12 HbA1c(period)

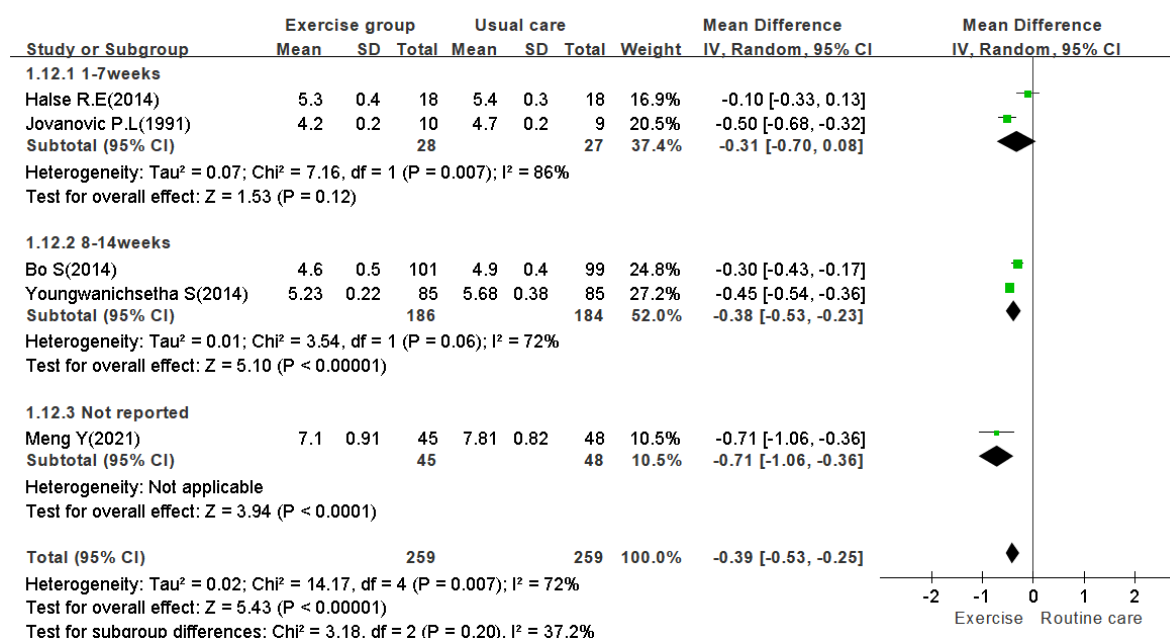

Supplementary Figure 8. Forest plot of the effect of duration for hemoglobin A1c.

Supplement: Supplementary Figure 8. — Forest plot of the effect of duration for hemoglobin A1c. [file whn-2025-08-25-1-Supplementary-Figure-8.pdf]
