# Supplementary material for: Effect of exercise interventions on glycemic control in women with gestational diabetes mellitus: a systematic review and meta-analysis
Source: Womens Health Nurs. 2025 Sep 30;31(3):176–91. doi: 10.4069/whn.2025.08.25.1 (PMC12571028; doi:10.4069/whn.2025.08.25.1)
Supplement: Supplementary Figure 2. — Forest plot of the effect of duration for fasting blood sugar. [file whn-2025-08-25-1-Supplementary-Figure-2.pdf]

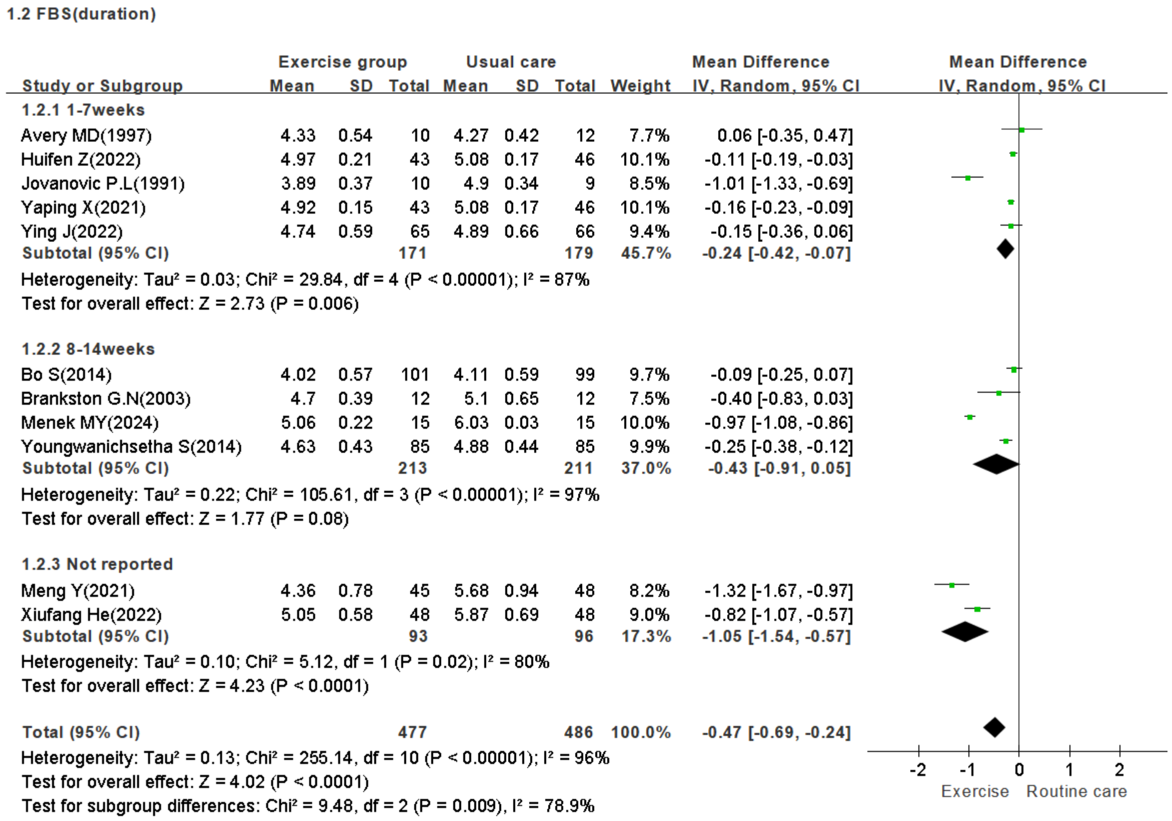

Supplementary Figure 2. Forest plot of the effect of duration for fasting blood sugar.

https://doi.org/10.4069/whn.2025.08.25.1
