# Supplementary material for: Effect of exercise interventions on glycemic control in women with gestational diabetes mellitus: a systematic review and meta-analysis
Source: Womens Health Nurs. 2025 Sep 30;31(3):176–91. doi: 10.4069/whn.2025.08.25.1 (PMC12571028; doi:10.4069/whn.2025.08.25.1)
Supplement: Supplementary Figure 4. — Forest plot of the effect of exercise type for fasting blood sugar. [file whn-2025-08-25-1-Supplementary-Figure-4.pdf]

1.4 FBS(type)

Study or Subgroup	Exercise group Mean	Exercise group SD	Exercise group Total	Usual care Mean	Usual care SD	Usual care Total	Weight	Mean Difference IV, Random, 95% CI
**1.4.1 Aerobic**								
Avery MD(1997)	4.33	0.54	10	4.27	0.42	12	7.7%	0.06 [-0.35, 0.47]
Bo S(2014)	4.02	0.57	101	4.11	0.59	99	9.7%	-0.09 [-0.25, 0.07]
Jovanovic P.L(1991)	3.89	0.37	10	4.9	0.34	9	8.5%	-1.01 [-1.33, -0.69]
Yaping X(2021)	4.92	0.15	43	5.08	0.17	46	10.1%	-0.16 [-0.23, -0.09]
**Subtotal (95% CI)**			**164**			**166**	**36.0%**	**-0.29 [-0.58, 0.00]**
Heterogeneity: Tau² = 0.07; Chi² = 28.80, df = 3 (P < 0.00001); I² = 90%								
Test for overall effect: Z = 1.94 (P = 0.05)								
**1.4.2 Resistance**								
Brankston G.N(2003)	4.7	0.39	12	5.1	0.65	12	7.5%	-0.40 [-0.83, 0.03]
Huifen Z(2022)	4.97	0.21	43	5.08	0.17	46	10.1%	-0.11 [-0.19, -0.03]
**Subtotal (95% CI)**			**55**			**58**	**17.6%**	**-0.18 [-0.41, 0.06]**
Heterogeneity: Tau² = 0.02; Chi² = 1.70, df = 1 (P = 0.19); I² = 41%								
Test for overall effect: Z = 1.45 (P = 0.15)								
**1.4.3 Others**								
Menek MY(2024)	5.06	0.22	15	6.03	0.03	15	10.0%	-0.97 [-1.08, -0.86]
Meng Y(2021)	4.36	0.78	45	5.68	0.94	48	8.2%	-1.32 [-1.67, -0.97]
Xiufang He(2022)	5.05	0.58	48	5.87	0.69	48	9.0%	-0.82 [-1.07, -0.57]
Ying J(2022)	4.74	0.59	65	4.89	0.66	66	9.4%	-0.15 [-0.36, 0.06]
Youngwanichsetha S(2014)	4.63	0.43	85	4.88	0.44	85	9.9%	-0.25 [-0.38, -0.12]
**Subtotal (95% CI)**			**258**			**262**	**46.5%**	**-0.69 [-1.10, -0.28]**
Heterogeneity: Tau² = 0.21; Chi² = 103.75, df = 4 (P < 0.00001); I² = 96%								
Test for overall effect: Z = 3.29 (P = 0.0010)								
**Total (95% CI)**			**477**			**486**	**100.0%**	**-0.47 [-0.69, -0.24]**
Heterogeneity: Tau² = 0.13; Chi² = 255.14, df = 10 (P < 0.00001); I² = 96%								
Test for overall effect: Z = 4.02 (P < 0.0001)								
Test for subgroup differences: Chi² = 4.53, df = 2 (P = 0.10), I² = 55.8%								

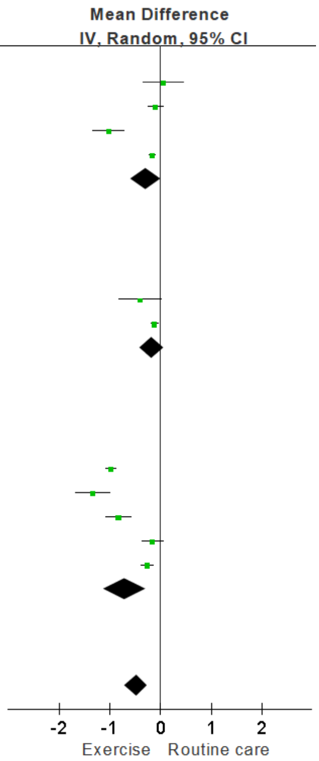

Supplementary Figure 4. Forest plot of the effect of exercise type for fasting blood sugar.
