# Supplementary material for: Effect of exercise interventions on glycemic control in women with gestational diabetes mellitus: a systematic review and meta-analysis
Source: Womens Health Nurs. 2025 Sep 30;31(3):176–91. doi: 10.4069/whn.2025.08.25.1 (PMC12571028; doi:10.4069/whn.2025.08.25.1)
Supplement: Supplementary Figure 5. — Forest plot of the effect of duration for 2-hour postprandial glucose. [file whn-2025-08-25-1-Supplementary-Figure-5.pdf]

1.7 PPG2hr(duration)

Study or Subgroup	Exercise group Mean	SD	Total	Usual care Mean	SD	Total	Weight	Mean Difference IV, Random, 95% CI
**1.7.1 1-7weeks**								
Avery MD(1997)	4.77	0.7	10	5.33	0.9	12	5.8%	-0.56 [-1.23, 0.11]
Huifen Z(2022)	6.06	0.22	43	6.25	0.22	46	12.8%	-0.19 [-0.28, -0.10]
Yaping X(2021)	6.11	0.11	43	6.25	0.22	46	12.9%	-0.14 [-0.21, -0.07]
Ying J(2022)	6.09	0.79	65	6.96	1.06	66	10.2%	-0.87 [-1.19, -0.55]
**Subtotal (95% CI)**			**161**			**170**	**41.6%**	**-0.33 [-0.53, -0.13]**
Heterogeneity: Tau² = 0.03; Chi² = 20.36, df = 3 (P = 0.0001); I² = 85%								
Test for overall effect: Z = 3.23 (P = 0.001)								
**1.7.2 8-14weeks**								
Bo S(2014)	5.89	1.05	101	6.5	0.92	99	10.8%	-0.61 [-0.88, -0.34]
Brankston G.N(2003)	5.7	0.4	12	6.2	1.1	12	5.9%	-0.50 [-1.16, 0.16]
Menek MY(2024)	8.23	0.64	15	9.05	0.38	15	9.4%	-0.82 [-1.20, -0.44]
Youngwanichsetha S(2014)	5.75	0.55	85	6.35	0.56	85	12.1%	-0.60 [-0.77, -0.43]
**Subtotal (95% CI)**			**213**			**211**	**38.2%**	**-0.62 [-0.76, -0.49]**
Heterogeneity: Tau² = 0.00; Chi² = 1.26, df = 3 (P = 0.74); I² = 0%								
Test for overall effect: Z = 9.38 (P < 0.00001)								
**1.7.3 Not reported**								
Meng Y(2021)	6.67	0.98	45	8.12	0.85	48	9.4%	-1.45 [-1.82, -1.08]
Xiufang He(2022)	6.91	0.62	48	7.62	0.76	48	10.8%	-0.71 [-0.99, -0.43]
**Subtotal (95% CI)**			**93**			**96**	**20.2%**	**-1.07 [-1.79, -0.34]**
Heterogeneity: Tau² = 0.25; Chi² = 9.70, df = 1 (P = 0.002); I² = 90%								
Test for overall effect: Z = 2.89 (P = 0.004)								
**Total (95% CI)**			**467**			**477**	**100.0%**	**-0.62 [-0.84, -0.40]**
Heterogeneity: Tau² = 0.09; Chi² = 106.99, df = 9 (P < 0.00001); I² = 92%								
Test for overall effect: Z = 5.61 (P < 0.00001)								
Test for subgroup differences: Chi² = 7.72, df = 2 (P = 0.02), I² = 74.1%								

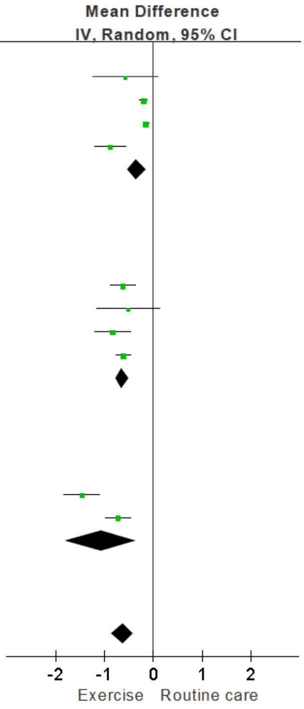

Supplementary Figure 5. Forest plot of the effect of duration for 2-hour postprandial glucose.
