# Supplementary material for: Effect of exercise interventions on glycemic control in women with gestational diabetes mellitus: a systematic review and meta-analysis
Source: Womens Health Nurs. 2025 Sep 30;31(3):176–91. doi: 10.4069/whn.2025.08.25.1 (PMC12571028; doi:10.4069/whn.2025.08.25.1)
Supplement: Supplementary Figure 6. — Forest plot of the effect of time for 2-hour postprandial glucose. [file whn-2025-08-25-1-Supplementary-Figure-6.pdf]

1.8 PPG2hr(time)

Study or Subgroup	Exercise group Mean	SD	Total	Usual care Mean	SD	Total	Weight	Mean Difference IV, Random, 95% CI
**1.8.1 15-29min**								
Bo S(2014)	5.89	1.05	101	6.5	0.92	99	10.8%	-0.61 [-0.88, -0.34]
Ying J(2022)	6.09	0.79	65	6.96	1.06	66	10.2%	-0.87 [-1.19, -0.55]
Youngwanichsetha S(2014)	5.75	0.55	85	6.35	0.56	85	12.1%	-0.60 [-0.77, -0.43]
**Subtotal (95% CI)**			**251**			**250**	**33.1%**	**-0.65 [-0.79, -0.51]**
Heterogeneity: Tau² = 0.00; Chi² = 2.24, df = 2 (P = 0.33); I² = 11%								
Test for overall effect: Z = 9.00 (P < 0.00001)								
**1.8.2 30-60min**								
Avery MD(1997)	4.77	0.7	10	5.33	0.9	12	5.8%	-0.56 [-1.23, 0.11]
Huifen Z(2022)	6.06	0.22	43	6.25	0.22	46	12.8%	-0.19 [-0.28, -0.10]
Yaping X(2021)	6.11	0.11	43	6.25	0.22	46	12.9%	-0.14 [-0.21, -0.07]
**Subtotal (95% CI)**			**96**			**104**	**31.5%**	**-0.16 [-0.22, -0.10]**
Heterogeneity: Tau² = 0.00; Chi² = 2.08, df = 2 (P = 0.35); I² = 4%								
Test for overall effect: Z = 5.43 (P < 0.00001)								
**1.8.3 Not reported**								
Brankston G.N(2003)	5.7	0.4	12	6.2	1.1	12	5.9%	-0.50 [-1.16, 0.16]
Menek MY(2024)	8.23	0.64	15	9.05	0.38	15	9.4%	-0.82 [-1.20, -0.44]
Meng Y(2021)	6.67	0.98	45	8.12	0.85	48	9.4%	-1.45 [-1.82, -1.08]
Xiufang He(2022)	6.91	0.62	48	7.62	0.76	48	10.8%	-0.71 [-0.99, -0.43]
**Subtotal (95% CI)**			**120**			**123**	**35.4%**	**-0.90 [-1.29, -0.51]**
Heterogeneity: Tau² = 0.11; Chi² = 11.69, df = 3 (P = 0.009); I² = 74%								
Test for overall effect: Z = 4.53 (P < 0.00001)								
**Total (95% CI)**			**467**			**477**	**100.0%**	**-0.62 [-0.84, -0.40]**
Heterogeneity: Tau² = 0.09; Chi² = 106.99, df = 9 (P < 0.00001); I² = 92%								
Test for overall effect: Z = 5.61 (P < 0.00001)								
Test for subgroup differences: Chi² = 50.02, df = 2 (P < 0.00001), I² = 96.0%								

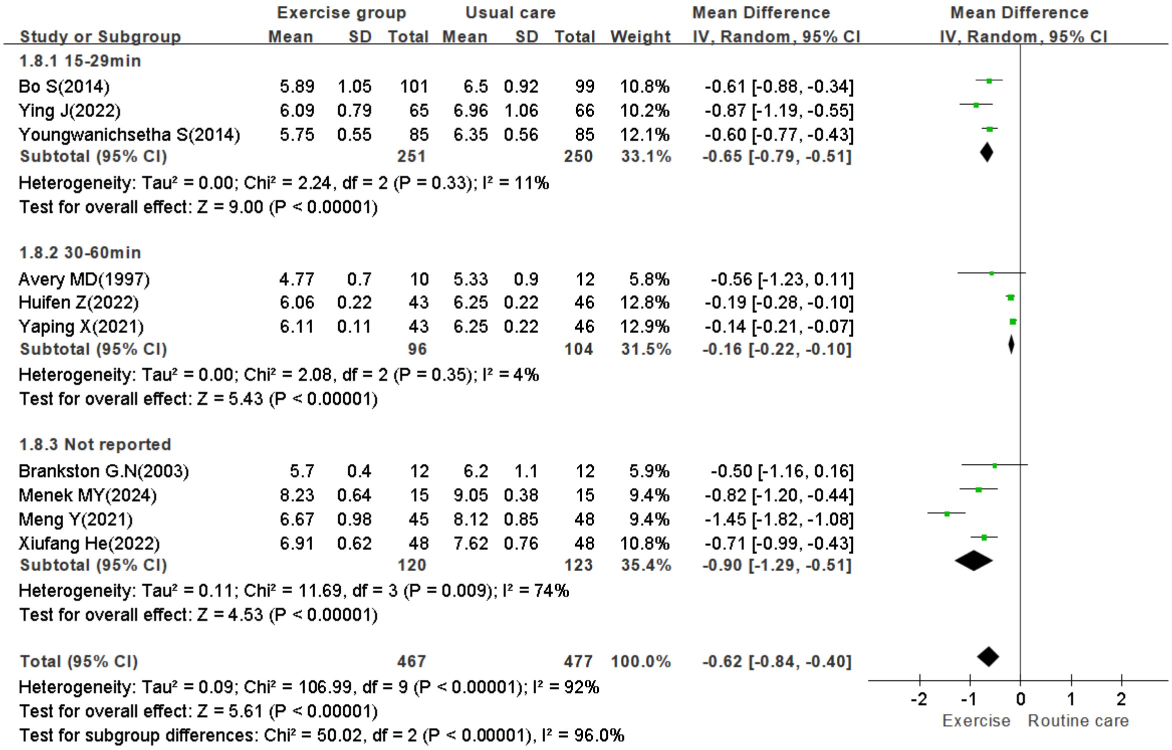

Supplementary Figure 6. Forest plot of the effect of time for 2-hour postprandial glucose.
